# Effect of Exercise Interventions on Irisin and Interleukin-6 Concentrations and Indicators of Carbohydrate Metabolism in Males with Metabolic Syndrome

**DOI:** 10.3390/jcm12010369

**Published:** 2023-01-03

**Authors:** Karol Makiel, Agnieszka Suder, Aneta Targosz, Marcin Maciejczyk, Alon Haim

**Affiliations:** 1 Department of Anatomy, Faculty of Physical Rehabilitation, University of Physical Education, 31-571 Cracow, Poland; 2 Department of Physiology, Faculty of Medicine, Jagiellonian University Medical College, 31-531 Cracow, Poland; 3 Department of Physiology and Biochemistry, Faculty of Physical Education and Sport, University of Physical Education, 31-571 Cracow, Poland; 4 Department of Endocrinology and Diabetes, Faculty of Health Sciences, Ben-Gurion University of the Negev, Beer-Sheva 653, Israel; 5 Soroka University Medical Center, Beer-Sheva 151, Israel

**Keywords:** irisin, interlekin-6, exercise, metabolic syndrome, obesity, physical activity

## Abstract

Irisin (IR) is a biomarker that is associated with metabolic syndrome (MetS). However, the available evidence on the association of IR, physical activity, and MetS status are contradictory. Therefore, the present study aimed to investigate the effect of exercise intervention on IR and interleukin-6 (IL-6) levels and indicators of carbohydrate metabolism in males with MetS. The study included 62 males with MetS (age 36.6 ± 6.9 years, BMI 33.6 ± 4.4 kg/m^2^) randomly assigned to: examined group 1 (EG1, n = 21) with aerobic exercise intervention, examined group 2 (EG2, n = 21) with combined aerobic and resistance exercise intervention, both for 12 weeks, and the control group (CG, n = 20) without intervention. Anthropometric measurements, body composition (body fat [BF], fat free mass [FFM]) as well as a biochemical blood analysis (irisin [IR], interleukin-6 [IL-6], insulin [INS] and glucose [GL]) were performed at baseline, 6 and 12 weeks of intervention, and 4 weeks after ending the intervention (follow-up). Intergroup and intragroup comparisons were performed. In EG1, an increase in IR level was observed as well as decreases in IL-6, BF, and GL levels in relation to the initial measurement. In EG2, decreases in IL-6, BF, and INS levels were observed as well as an increase in FFM level. In CG, no changes were found. Aerobic-resistance exercise led to a greater reduction in the concentrations of IL-6 and INS and more favorable changes in body composition (BF and FFM) than the use of aerobic training alone in males with MetS.

## 1. Introduction

Metabolic syndrome (MetS) is characterized by the occurrence of a few mutually connected disorders of metabolic character, including insulin resistance, atherogenic dyslipidemia, visceral obesity, and hypertension. MetS is also linked with chronic inflammation of low intensity. Untreated MetS is related to increased risk of developing diabetes and cardiovascular diseases (CVD) [1].

The pathophysiology of MetS comprises several complex mechanisms that have not been fully explained. MetS can be caused by genetic and epigenetic determinants [2]. The development of MetS is enhanced by a lifestyle combining a low level of physical activity with a high dietary intake of energy that exceeds the needs of the organism [3,4,5].

It has been demonstrated that myokines such as irisin (IR) and interleukin 6 (IL-6) are released through skeletal muscles during physical activity. They induce positive physiological and metabolic effects not only in skeletal muscle but also in distant tissues, such as white adipose tissue, the liver, bones, immune system cells, and the central nervous system, thus decreasing inflammation, improving the sensitivity of cells to insulin, and correcting energy expenditure [6,7,8].

IR is a myokine produced mainly in skeletal muscle during physical effort, and, to a lesser extent, by white adipose tissue [9]. In mice, IR originating from muscle accounts for ~72% of the total level of the hormone in circulation, and the remaining 28% probably originates in adipose tissue [10]. The main effect of IR is the browning of white adipose tissue, an increase of energy expenditure of the body, improvement of cell sensitivity to INS, and body mass reduction [11]. IR stimulates lipolysis and increases the release of GL and free fatty acids during physical activity [12]. Higher concentrations of IR are observed in obese patients [13]. Moreover, IR positively affects lipid disorders that result from obesity and MetS [14]. In a meta-analysis of 18 studies, higher concentrations of IR were registered in overweight or obese people in relation to people with proper body weight, which can be explained by the phenomenon of ‘IR resistance’ [15]. A similar process has been observed in patients with MetS [16]. The application of resistance training leads to a higher production of IR than moderate intensity training or interval exercises of high intensity. The increase in IR production is detectable immediately after the beginning of training and its stabilization takes place up to one hour after the exercise is finished. The presented dependencies relate both to people with MetS and healthy individuals [17].

IL-6 is a cytokine of which increased concentrations secreted by adipocytes and monocytes are thought to be responsible for inflammation and INS resistance in obesity. However, it was demonstrated that IL-6 released by skeletal muscle features an anti-inflammation effect and increases sensitivity to INS [18]. The concentrations of IL-6 and IR and parameters of carbohydrate metabolism, such as HOMA, were dependent on the level of adipose tissue and increased with the degree of obesity in examined patients [19]. The production of IL-6 is related to the level of glycogen and the intensity and duration of physical activity [20]. The increase in IL-6 level can be higher after moderate intensive exercises with long duration (i.e., running), which engage many muscle parties, than after isolated resistance training [21].

Physical activity leads to many endocrine interactions between skeletal muscles, adipose tissue, and other organs of internal secretion. The introduction of regular physical activity results in changes in the concentrations of circulating myokines, adipokines, and immunological cytokines and the subsequent reduction of body mass, a decrease in the inflammatory condition, INS resistance, and other disorders associated with MetS [22,23,24]. Aerobic training leads to a significant increase in energy expenditure and creates advantageous conditions to decrease excessive adipose tissue mass, whereas resistance training is of significant importance to increasing fat free body mass, which results in higher INS sensitivity and efficiency in maintaining and increasing the resting metabolic rate [25]. The introduction of regular resistance exercises combined with endurance exercises prevents the recurrence of obesity [25] and improves indices of MetS [26].

The significant role of physical training in improving health conditions can result in a decrease of IR and IL-6 concentrations in people with MetS. The applied training may cause a decrease in the visceral fat level and improvement of MetS parameters. Knowledge of the differences and benefits of applied training may allow males with MetS to choose the most favorable form of intervention to improve their health. The aim of this research was to assess the effects of twelve weeks of two different exercise interventions (training of an aerobic character vs. combined aerobic-resistance training) and four weeks of follow-up on the concentrations of IR and IL-6 in males with MetS. We hypothesized that combined aerobic-resistance exercise intervention would positively affect the secretion of adipocytokines while having anti-inflammatory effects in males with MetS.

## 2. Materials and Methods

### 2.1. Materials

The study design was a prospective, randomized, and controlled trial to investigate the effects of two types of twelve-week exercise intervention (training of an aerobic character vs. combined aerobic-resistance training) on body composition, levels of selected adipokines, and indices of metabolic syndrome (MetS) in males with MetS in relation to males with MetS who did not participate in training (control group, CG). The examined participants from all three groups were then observed for a four-week follow-up period (without scheduled training).

**Figure 1:**
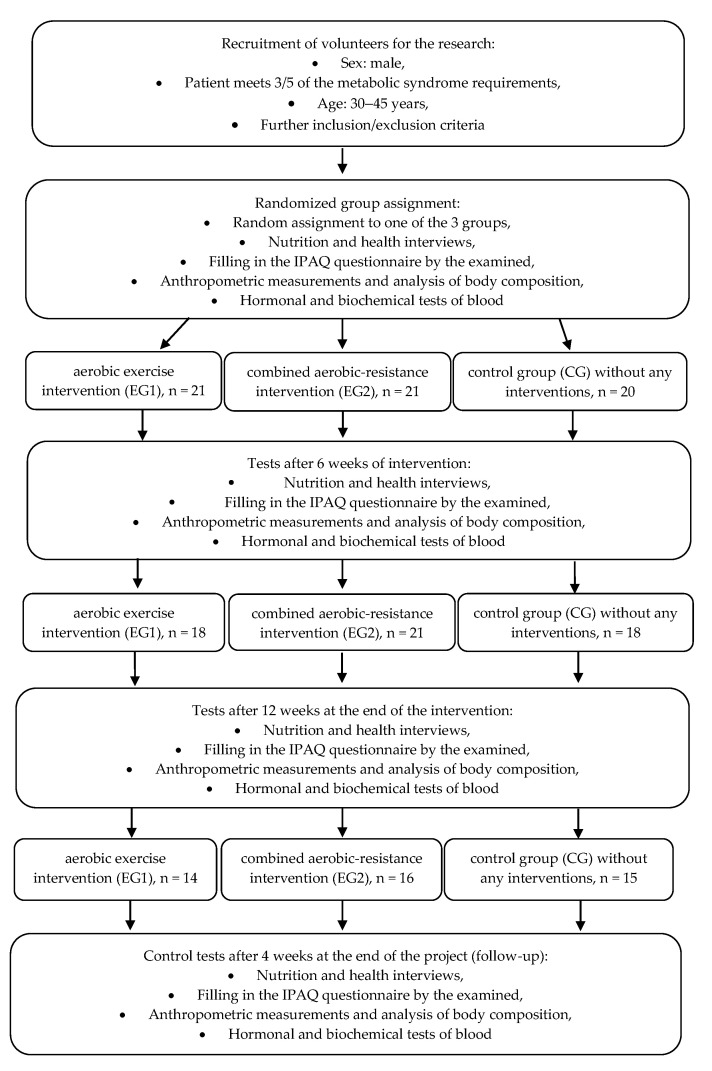
The course of the study.

Due to the character of the intervention, a blind trial was not applied; however, the lab personnel, biostatistician, and analysis team were not aware of the group assignments. The studies were registered in the clinical trials registry on the ANZCTR platform (Australian New Zealand Clinical Trials Registry): ACTRN registration number 12622001394730. The course of the study is presented in Figure 1.

The study included 62 Caucasian males aged 30–45 (mean age 37 ± 7) years with waist circumferences ≥ 94 cm (visceral obesity is a prerequisite for the diagnosis of MetS) and with two out of four criteria of MetS: concentration of triglycerides > 150 mg/dL (1.7 mmol/L) or hypertriglyceridemia under treatment; concentration of HDL C < 40 mg/dL (1.03 mmol/L) or this lipid disorder under treatment; systolic blood pressure (SBP) ≥ 130 mm Hg or diastolic (DBP) ≥ 85 mm Hg, or treatment of formerly diagnosed hypertension; fasting level of GL in blood plasma ≥ 100 mg/dL (5.6 mmol/L) or pharmacological treatment of diabetes type 2 (IDF, International Diabetes Federation, 2006 [27]). The participants were assigned randomly into 3 groups; qualification was based on simple randomization following sealed opaque envelopes:

1. Experimental group 1 (EG1) of males with MetS (n = 21) performing aerobic exercise intervention;
2. Experimental group 2 (EG2) of males with MetS (n = 21) performing combined aerobic-resistance exercise intervention;
3. Control group (CG) of males with MetS (n = 20) not undertaking any physical activity.

The study inclusion criteria were: male aged 30–45 years, medical statement on lack of contraindications to take up health training of aerobic-resistance character, written consent for voluntary participation in the research project, waist circumference ≥ 94 cm, and two criteria out of the following: concentration of triglycerides > 150 mg/dL (1.7 mmol/L) or treated hypertriglyceridemia; concentration of HDL C < 40 mg/dL (1.03 mmol/L) or treatment of the lipid disorder; blood pressure systolic (SBP) ≥ 130 mm Hg or diastolic (DBP) ≥ 85 mm Hg, or treatment of formerly diagnosed hypertension; GL level in plasma on empty stomach ≥ 100 mg/dL (5.6 mmol/) or pharmacological treatment of type 2 diabetes (T2DM). In EG1, the presence of chronic diseases was confirmed, such as: hypertriglyceridemia (8 people), arterial hypertension (12), hypercholesterolemia (4), T2DM (5), chronic sinusitis (1), gout (2), and psoriasis (2). Chronic diseases were confirmed in EG2: hypertriglyceridemia (13), arterial hypertension (15), hypercholesterolemia (9), T2DM (2), and gout (1). In CG, the following chronic diseases were confirmed: hypertriglyceridemia (13), arterial hypertension (15), hypercholesterolemia (5), T2DM (4), and chronic sinusitis (1).

The exclusion criteria were: no medical statement on lack of contraindications to take up health training of aerobic-resistance character, unwillingness to continue intervention (more than 10% of training sessions missed), unstable ischaemia, decompensated heart failure, uncontrolled heart rhythm disorder, severe pulmonary hypertension (mean blood pressure in lungs > 55 mm Hg), symptomatic aortic stenosis, acute myocarditis, endocarditis or pericarditis, uncontrolled blood pressure (>180/110 mm Hg), aortic dissection, Marfan syndrome, uncontrolled diabetes, mental disorders, health problems (orthopedic, neurologic) preventing movement, participation in another form of physical activity during the project, and lack of written consent to take part in the examination.

The volunteers were informed about the procedures and purpose of the research in detail and about the possibility of resigning from participation at any stage. During the period of intervention, there were resignations from the study. The reasons for excluding a participant from the project included: absenteeism at more than 10% (6), excessive alcohol consumption (2), absence during control visits (3), and occurrence of COVID-19 symptoms (3). The number of participants required to show statistical significance was based on previously published studies in this field. An error probability (α) of 0.05, power (1 − β) of 0.80, and an average effect size (d) of 0.8 were used to calculate the sample size. All the examined participants were asked not to alter their nutritional habits, taken medicines, or level of physical activity during the observation. All volunteers provided written consent for participation in the study, as well as for the use of their personal data and research results for scientific purposes. The research project obtained the approval of the Ethics Committee of the Regional Medical Chamber in Krakow (90/KBL/OK/2020).

### 2.2. Methods

For all participants, he following assessments were performed 4 times: before the intervention, in the middle after 6 weeks of intervention, after 12 weeks of intervention, and 4 weeks after ending the intervention (follow-up period):

#### 2.2.1. Anthropometry

For the purpose of this study, body height (BH) [cm], body mass (BM) [kg], and waist circumference (WC) [cm] were used. BH was measured to the nearest 1 mm, in a standing position without shoes, with the head in the Frankfurt plane, using a stadiometer (Seca 231 stadiometer, Hamburg, Germany). BM was obtained in the standing position using a standardized medical scale (Beurer PS 240, Budapest, Hungary), with an accuracy of 50 g. WC was measured to the nearest 1 mm using an anthropometric tape between the lower edge of the costal arch and the upper edge of the iliac crest, with the participant in standing position, and recorded at the end of a gentle expiration.

#### 2.2.2. Body Composition

Dual-Energy X-ray Absorptiometry (DEXA] was applied to assess body composition: percentage of body fat (BF) [%], fat free mass (FFM) [kg], and body mass index (BMI) [kg/m^2^].

Assessment of body composition was carried out using the Lunar Prodigy Primo PR+352163 (Chicago, IL, USA) device according to the manufacturer’s guidelines.

#### 2.2.3. Hormonal Blood Indices

Fasting blood samples were collected in the morning after a 24-h break from training, from the basilic, cephalic, or median cubital vein into test tubes (Vacumed^®^ system, F.L. Medical, Torreglia, Italy) by the experienced nursing team. The collected blood was centrifuged (RCF 1000× *g*) immediately after collection for 15 min at 4 °C (MPW-351R, MPW Med. Instruments, Warsaw, Poland), and the serum was collected and stored at −80 °C until further study (BIO Memory 690L, Froilabo, Paris, France).

The concentrations of IR and IL-6 were measured using commercially available ELISA kits according to the manufacturer’s protocol. The human IR ELISA Kit (catalogue number 201-12-5328) was purchased from Shanghai Sunred Biological Technology Co. (Shanghai, China). The IL-6 ELISA kit (catalogue number IL E-3200IL-6) was purchased from LDN Labor Diagnostika Nord GmbH & Co.KG (Am Eichenhain, Germany). An ELx 808 spectrophotometric microplate reader (BioTek, Winooski, VT, USA) was used to determine the optical density at 450 nm. Marking was performed at the Laboratory of Genetics and Molecular Biology, Department of Physiology, Jagiellonian University Medical College, Kraków, Poland.

#### 2.2.4. Biochemical Blood Indices

The concentration of GL [mmol/L] in the blood plasma was performed via the enzymatic method using the Cobas c701/702 biochemical analyzer (Roche Diagnostics International Ltd., Mannheim, Germany). The serum insulin [µIU/mL] concentration was determined by electrochemiluminescence (ECLIA) using the Cobas e801 apparatus (Roche Diagnostics International Ltd., Mannheim, Germany). The determinations were performed according to manufacturer’s guidelines with the use of reagents dedicated to the GLUC3 and Elecsys Insulin analyzers, respectively.

#### 2.2.5. Evaluation of Energy Expenditure and Energy Value of Diet

The International Physical Activity Questionnaire (IPAQ) was conducted to assess daily energy expenditures [28]. The total energy expenditure [kcal/week) was calculated based on the sum of non-exercise activity thermogenesis (NEAT) assessed based on the IPAQ questionnaire and energy expenditures connected with intervention in groups EG1 and EG2.

A qualified dietician carried out a 24-h nutrition interview using a nutrition record to quantitatively assess nutrition habits and monitor alterations in the diet during training. The results were introduced into the DietaPro program version 4.0 (Institute of Food and Nutrition, Warsaw, Poland). The energy value of the diet was assessed as kcal/week.

### 2.3. Exercise Interventions

The exercise interventions took place at a fitness club in Cracow under supervision of a personal coach. All of the training was carried out at the same time of day (evening, 6–9 pm) by the same personal coach, in a room with the same temperature (22 degrees Celsius) and humidity. A session attendance checklist was used to monitor adherence to the intervention. Examined participants who attended less than 90% of the training sessions for 12 weeks were eliminated from the observation and statistics.

Planning and monitoring of the intensity of the aerobic training as well as the amount of load in the resistance training was set individually based on the guidelines of the American College of Sports Medicine [29]. Heart rate (HR) during training was monitored using the Polar M200 GPS Running Watch with Wrist-Based Heart Monitor (Kempele, Finland). Before the resistance training, the One Repetition Maximum (1 RM) was determined. The examined participants underwent the 1 RM test before the examination, and after 6, 12, and 16 weeks. The personal coach carried out the warm-up on the treadmill (Technogym New Excite Run Now 500, Cesena, Italy) for 5 min at 60% HR. Next, the participant started resistance training. The last repetition of a series occurred when the participant could not continue to exercise maintaining the proper technique. The obtained load and number of repetitions were converted into 1 RM based on the 1 RM calculator [30].

The aim of the intervention was to realize 3 training sessions per week, which were converted into 3 × 6 MET of energy expenditure for a week interchangeable with running, and 3 × 5.5 MET for resistance training (Compendium of Physical Activities, 2011) [31].

#### 2.3.1. Aerobic Training

The course of the aerobic intervention: training sessions took place 3 times per week in groups of max 5 participants, starting with a 5-min warm-up (treadmill walk, Technogym New Excite Run Now 500, Cesena, Italy), reaching 50% maximal heart rate (HR max). After the warm-up, the participants increased intensity to 70% HR max, based on increasing velocity or angle for a tread-mill, resistance for upright bikes (Technogym Artis, Cesena, Italy), and range of movement or resistance for an x-trainer (Precor EFX556i Elipsa, Woodinville, WA, USA). Aerobic exercises were performed mainly on a treadmill (fast walk or jog). When the examined participant reported muscle pain, they could use a different training device (upright bike or x-trainer) to reduce discomfort and lower the chance for injury. The training was of a continuous character with a steady HR. The duration of the aerobic training was 45 min. After the training, the participants stretched the engaged muscle groups for 10 min.

#### 2.3.2. Combined Aerobic-Resistance Training

The course of the aerobic-resistance intervention (Table S1, Supplement): the training with elements of aerobic-resistance exercises took place 3 times per week in groups of max 5 participants. The training started with a 5-min aerobic warm-up: walking on a treadmill (Technogym New Excite Run Now 500, Cesena, Italy) to reach an intensity of 50% HR max. Initially, the training comprised 3 complex exercises involving the whole body (FBW—full body workout), such as squats, push-ups with adjustable height of arm prop, bent isolated one-arm dumbbell row, 4 series with 120-s breaks between them. In the second week of intervention, following adaptation of the body to the training, the training changed to 3 series of 6 exercises, with 90-s breaks between them. From the third week of intervention, the training included 3 series of 9 exercises, with 60-s breaks between them. At first, the load was set at 50% of one repetition maximum (1 RM), then it was raised to 70% 1 RM after 4 weeks of training. After the resistance exercises, the participants were trained on the treadmill (Technogym New Excite Run Now 500, Cesena, Italy), upright bike (Technogym Artis, Cesena, Italy), or x-trainer (Precor EFX556i Elipsa, Woodinville, DC, USA) at an intensity of 50% HR max in the first week and 70% HR max from the second week of intervention. The duration of the resistance training sessions was 30, 35, and 40 min, respectively, followed by 20, 15, and 10 min for aerobic training, respectively. Training finished with stretching the engaged muscle groups for 5 min. The duration of the whole training was 60 min. The training engaged large muscle groups, whereas in subsequent exercises, through isolation of the practised exercise positions, the synergistic muscles of lower mass were activated. The training employed dumbbells, barbells, training devices, and the participant’s own body mass. The progression of load (%) in the selected resistance exercises during the intervention and follow-up, calculated based on the data obtained in the force test 1 RM [32], compared to baseline are presented in Table 1 for EG2.

### 2.4. Statistical Analysis

The distribution of results for the analyzed variables was checked by applying a Shapiro–Wilk test. Due to a skewed distribution of most variables, the differences between the study groups and the control group were assessed using the Kruskal-Wallis test.

To compare the impact of intervention on changes in the analyzed variables between EG and CG, the Friedman test with post hoc comparison (Wilcoxon-Nemenyi test) was used. The effect size (E.S.) was estimated for the Friedman test: W = X2/N(K − 1); where W is the Kendall’s W value, X2 is the Friedman test statistic value, N is the sample size, and K is the number of measurements per subject. The Kendall’s W coefficient assumes a value from 0 (indicating no relationship) to 1 (indicating a perfect relationship). Kendalls uses the Cohen’s interpretation guidelines of 0.1 ≤ 0.3 (small effect), 0.3 ≤ 0.5 (moderate effect) and ≥0.5 (large effect) [33]. The Spearman correlation coefficient (r) was calculated.

In order to explain the variability in IL-6 and IR levels, multiple regression was applied. The models were prepared using an econometric linear model of multiple regression assessed by the method of least squares. In both models, the residual standard errors and test *p*-values were corrected using heteroscedasticity-adjusted robust standard errors.

In all analyses, the effects were assumed significant if their probability value *p* was lower than the accepted significance level α = 0.05 (*p* < 0.05).

The ggplot2 package of RStudio IDE in R programming language was used to perform all calculations.

## 3. Results

There were no differences between EG1, EG2, and CG with respect to age, number of parameters of MetS confirmed in the examined males, and basic anthropometric parameters before the interventions (Table 2).

After applying the health training interventions both in EG1 (*p* = 0.00) and EG2 (*p* = 0.00), an increase in energy expenditure [kcal/week] was confirmed at each week of measurements in relation to the first week of tests. In CG, the total energy expenditure did not change significantly through the whole period of observation. The energy value of the diet during the intervention after 6 (*p* = 0.53) and 12 (*p* = 0.22) weeks did not change in EG1, but a remarkable increase in delivered calories was noticed during the follow-up period (*p* = 0.01) in the diet of the described group. Similarly, an increase in delivered calories in the diet was confirmed in EG2 (*p* = 0.00). In CG, the energy value of the diet during the observation period did not change significantly (*p* = 0.35) (Table 3).

After applying 6 (*p* = 0.00) and 12 (*p* = 0.00) weeks of aerobic-resistance exercise intervention, a significant (*p* = 0.00) increase in FFM was confirmed in the examined males (EG2). In contrast, no change in FFM was found in either EG1 or CG. Changes in total percentage of BF were found in EG1 (*p* = 0.05) and EG2 (*p* = 0.01) at 6, 12, and 16 weeks compared to the initial measurements. The highest decrease in BF level was observed in EG2 (Table 4), while a increase in BF level was confirmed between measurements at 6 and 12 weeks in CG (data not included).

Significant changes (*p* = 0.03) in the level of INS were observed between measurements in EG2. A decrease in INS level took place between 1 and 16 weeks (*p* = 0.03). A significant change in the concentration of GL was found in a single measurement: a decrease in GL concentration was confirmed between measurements at weeks 1 and 16 in EG1 (*p* = 0.02) (Table 5).

**Figure 2:**
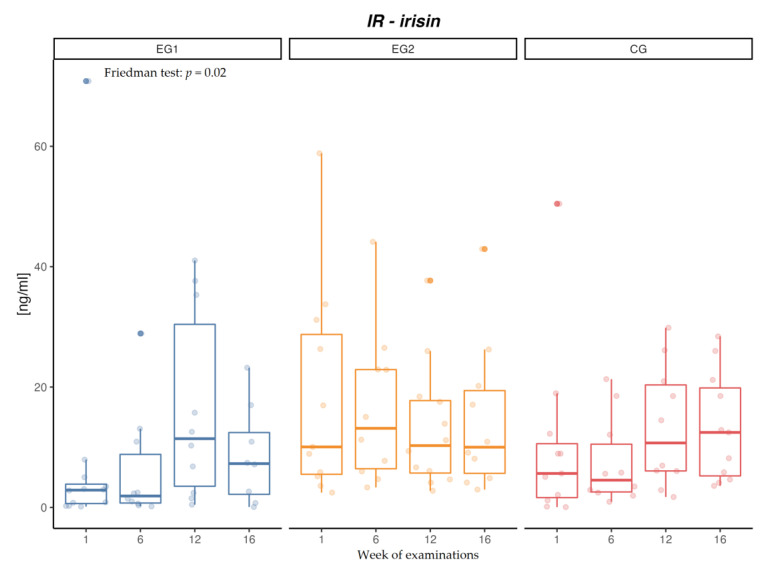
Changes in irisin (IR) concentration [ng/mL] in the aerobic group (EG1), aerobic-resistance group (EG2), and control group (CG) during weeks of examinations.

**Figure 3:**
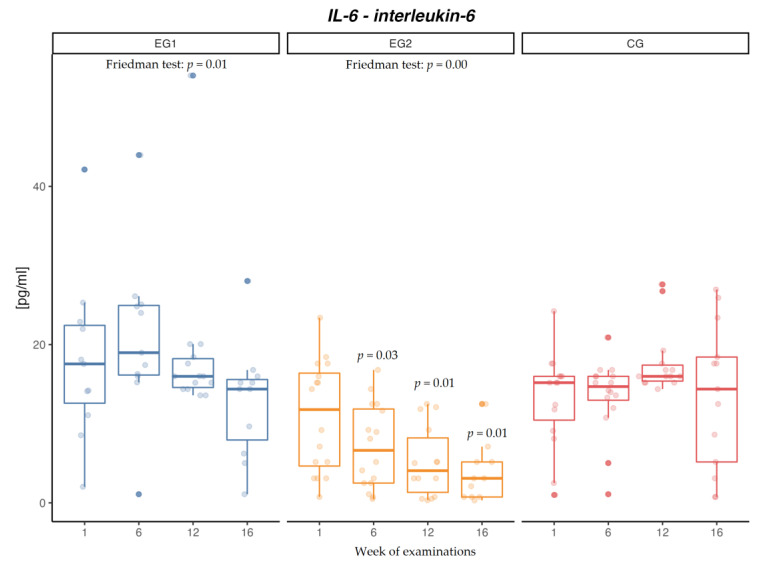
Changes in interleukin-6 (IL-6) concentration [pg/mL] in the aerobic group (EG1), aerobic-resistance group (EG2), and control group (CG) during weeks of examinations.

After applying aerobic exercise intervention in the examined males (EG1), an increase in IR concentration was confirmed (*p* = 0.02) (Figure 2). After aerobic-resistance exercise intervention (EG2), no changes were found in the concentration of IR, both after 6 and 12 weeks of intervention and after the follow-up period. In the control group (CG), no changes were found in the concentration of IR. After applying exercise intervention in both EG1 (*p* = 0.01) and EG2 (*p* = 0.01), a decrease in the concentration of IL-6 was found, and mean concentrations of IL-6 were lower at each subsequent measurement in EG2 (Figure 3). Intergroup differences were confirmed after 6 (*p* = 0.00) and 12 (*p* = 0.00) weeks between EG1 and EG2 as well as between EG2 and CG, and after 16 weeks (*p* = 0.01) between EG1 and EG2 and between EG2 and CG. In the control group (CG), no changes were found in the concentration of IL-6 (Table 6).

Significant correlations (Table 7) were confirmed in EG1 between IR and energy expenditure (r = 0.27), FFM (r = 0.35), concentration of GL in blood (r = 0.44), INS (r = 0.37), and also between IL-6 and energy expenditure (r = 0.35), FFM (r = 0.44), GL (r = 0.31), and INS (r = 36). In EG2, correlations were found between the concentrations of IR and IL-6 (r = −0.31) and energy value of the diet (r = 0.27), as well as between IL-6 and energy expenditure (r = −0.35) and BF (r = 0.38). In CG, correlations were observed between IR and BF (r = −0.30), GL (r = −0.36), and INS (r = −0.42). No correlations were found between IL-6 and other parameters in CG (Table 7).

The applied multiple regression model demonstrated that both BF and FFM were significantly connected with the concentration of IR (*p* < 0.05). Males in EG2 featured significantly lower concentrations of IR than males in EG1. The analyzed variables explained 14% of variability in IR (value of R^2^ model = 0.14) (Table 8).

The applied multiple regression model showed that BF, FFM, and the type of examined group together explained 32% of the variability in IL-6 (value of R^2^ model = 0.32). Males in EG2 and CG were characterized by a significantly higher concentration of IL-6 than males in EG1 (Table 9).

## 4. Discussion

The main aim of the research was to assess the effects of 12 weeks of two different exercise interventions and 4 weeks of follow-up on the concentrations of IR and IL-6 in males with MetS.

While interpreting fluctuations in the concentrations of IR and IL-6, the moment of measuring the concentration of hormones in plasma should be taken into account. In this study, the measurements were taken at fasting 24 h after training. Under such conditions, the increased concentrations of Il-6 and IR may have resulted from increased synthesis in the adipose tissue. IL-6 and IR levels increase mainly under the influence of effort and skeletal muscle activation, and their measurement should be performed on the training day, preferably during training or immediately after finishing it [34,35]. It was demonstrated that IR levels instantly increase after the beginning of physical activity in adults with and without MetS [17,36,37]. The response of IR release depends on the intensity of the effort. Higher levels are observed after more intensive effort [38]. The concentration of circulating IL-6 increases exponentially in response to physical activity [34]. Concentrations of IL-6 in plasma may increase by 100 times in response to exercise [21]. The concentration of the hormone stabilizes 24 h after exercise intervention [35].

There are contradictory reports on the results of IR concentrations connected with long-term (≥8 weeks) intervention of physical activity. When both resistance and aerobic training were applied to groups with either normal body mass or obesity, the authors of a meta-analysis observed an increase in IR concentration and long-term results were obtained by people performing resistance training [39]. Additionally, in the study by Cosio et al. [40], an increased concentration of IR was found in people performing resistance training. In the meta-analysis by Qiu et al. [41], the authors registered a decrease in IR level in aerobic training intervention groups. Meanwhile, in an overview of studies on healthy adults, no significant changes were observed in the concentration of IR after performing long-term resistance or aerobic training [42].

IR is mainly produced in the tissue of skeletal muscles under the influence of physical effort, but it is also synthesized in adipocytes [43]. The occurrence of the ‘IR resistance’ phenomenon in patients with MetS and in an obese population was previously reported [16]. Another cause of the increased concentration of IR in the blood of obese patients may be the higher secretion of the hormone from adipose tissue [44]. A positive correlation between IR concentration and body mass, BMI, fat mass, and INS resistance was confirmed by Perakakis et al. Despite the fact that the concentration of IR was higher in obese patients, it was lower in patients with type 2 diabetes (T2DM) in some studies [45]. In the research of Norheim et al. [46], a 12-week training intervention decreased the concentrations of IR both in groups of healthy males and in those with INS resistance. Taking into consideration fluctuations of hormones connected with the issue of resistance, this study was expected to show increased concentrations of IR in the participants’ blood and decreased levels influenced by intervention, correlated with the loss of body fat and an increase in fat free body mass. In the group with aerobic training, after 6 weeks of intervention, there was a decrease in the percentage of BF and a decrease in IR concentration, whereas no changes were observed in the level of FFM. The increased IR concentrations in EG1 could have resulted from changes associated with the physical exercise effect (energy expenditure, r = 0.27). IR released from muscles stimulates increased mRNA expression of uncoupling protein 1 (UCP1) in adipocytes, leading to the transformation of white adipose tissue into brown adipose tissue, causing energy expenditure and inducing thermogenesis and GL homeostasis. In this group, the concentration of GL gradually decreased and a significant decrease took place 16 weeks after beginning the intervention. In the aerobic group, a positive correlation between IR concentration and fat free mass was also confirmed. A positive correlation between IR concentration and muscle mass or FFM was also observed by other scientists [37,47,48]. The mechanisms underlying the positive energy balance in obese patients are numerous, complex, and multifactorial. They exist largely beyond conscious control and are only partially the same among patients [49].

In the aerobic-resistance group, no significant changes in IR concentration were observed after 12 weeks of intervention. However, a significant decrease in the percentage of adipose tissue (−6.9%) and increase in the percentage of FFM (6.0%) were confirmed, despite an increase in the energy value of the diet in the analyzed period of time. A possible cause of the increased energy value of the diet was an increase in the feeling of hunger among examined participants after introducing physical activity and higher energy expenditures.

In this study, the mean level of BMI was 33.63 kg/m^2^ and the participants met at least 3 of 5 of the criteria of MetS. According to a previously published report [15], such a group has a high risk of IR resistance; therefore, a beneficial effect obtained through intervention is the lowering of IR concentrations, as found in EG1 during the first 6 weeks of observation. In a previous paper [13] investigating the relationship between IR and metabolic diseases, including obesity, type 2 diabetes, and hepatic steatosis, different test results were observed. It was shown that the concentration of IR was high in a group of obese people, whereas patients with type 2 diabetes and hepatic steatosis had lower concentrations of IR in relation to the control group. Researchers observed higher concentrations of IR and IL-6 in obese males with a tendency to suffer from MetS [19].

In this study, a gradual decrease in IL-6 concentrations was observed in the groups performing physical activity: in the aerobic-resistance group it amounted to −74% after 16 weeks and −18% was observed in the aerobic group. In the control group, no significant changes were found. The difference between groups indicated lower concentrations of IL-6 in the group performing aerobic-resistance training. The pathophysiology of obesity includes the occurrence of a chronic inflammatory condition with low intensity as a result of, among other factors, excessive synthesis of IL-6 by adipose tissue [50]. In the present work, a positive correlation was found between the percentage of adipose tissue and IL-6 level in EG2. Increased concentrations of IL-6 occur in patients with obesity and type 2 diabetes [51]. Myokine IL-6 is of significant importance to muscle efficiency during a muscle contraction, while IL-6 synthesized in adipose tissue, especially when it is chronically increased, may lead to INS resistance in muscles. Despite the fact that IL-6 synthesized in muscles provides therapeutic potential for INS resistance, there appear to be challenges connected to distinguishing the source of IL-6 synthesis, i.e., adipose tissue or skeletal muscle [52,53]. Observing a significant decrease in INS concentrations in EG2, accompanied by the highest decrease in IL-6 concentrations and the highest increase in FFM level, suggests that the changes result from the decrease in adipose tissue content, leading to lowering of the inflammatory condition in the body and INS resistance connected with obesity. The presented multiple regression model confirmed that BF and FFM explained 14% of the variability in IR concentration and 32% of the IL-6 variability. The present work demonstrated a significant negative correlation between the concentrations of IR and IL-6 in EG2. IR features anti-inflammatory properties that can lead to the decreased secretion of inflammatory cytokines, such as TNF-alpha and IL-6 [54].

There is information on the relationship between IR, IL-6, and the metabolism of carbohydrates in previous reports; therefore, an analysis of GL and INS concentrations was performed at individual timepoints in the current study. In EG1 and CG, no changes in the concentration of INS were observed. In EG2, after 6 weeks of intervention, an increase in INS concentration was confirmed, and a significant decrease was found in subsequent measurements that amounted to −34% after 16 weeks. In the post-hoc test, a significant decrease in GL level was registered after 16 weeks of intervention in EG1 and there was also a change between 6 and 16 weeks of intervention in EG2 (data not included). The relationship between IR and INS resistance is not explicit. In the study by Park et al. [15], it was observed that the concentration of IR was higher in people with MetS and was closely connected with INS resistance. However, in the research by Choi et al. [55], it was found that concentrations of IR were lower in people with type 2 diabetes and no relation was observed between IR and INS resistance.

IR leads to the browning of adipose tissue, which may affect the metabolism of GL. In humans, brown adipose tissue features ten times higher GL uptake through INS after exposure to cold than white or visceral adipose tissue [56]. Moreover, IR induces the expression of glucotransporter 4 (GLUT4) in mature human adipocytes [57]. Introduction of recombinant IR to human skeletal cells significantly increased GL and fatty acid uptake (30–40%) for approximately 1 h. Such intervention provided similar results as the uptake observed after exposure to INS [58]. In humans, the synthesis of FNDC5 and secretion of IR are higher in the skeletal muscles of people with obesity but without T2DM, probably to maximize GL uptake by muscles and prevent hyperglycemia [59,60]. There are reports that INS may not have an influence on the secretion of IR, regardless of the concentration, in people with INS resistance or obesity. In people with T2DM, GL is an important regulator of IR secretion from skeletal muscle [60].

This study was not free from limitations. The focus of this study was to comprehensively investigate clinical and/or functional adaptations in a relatively non-invasive manner, whereas assessment of the detailed molecular mechanisms was not possible. The monitoring of physical activity was conducted using devices monitoring heart rate; however, more precise supervision could be obtained by measuring VO2 max [61]. The examined participants increased the energy value of their diet despite registering their dietary habits and recommendations to keep their previous nutritional standards. This study only included male participants, and finally, the group sample sizes were relatively small and as such, added to variability in our data.

In summary, it should be emphasized that the application of combined aerobic-resistance training led to a higher decrease in IL-6 and INS concentrations and advantageous changes in the body composition compared to performing only aerobic training in males with MetS.

## Figures and Tables

**Table 1:** The progression of load [%] in selected resistance exercises during the intervention and follow-up compared to baseline in the aerobic-resistance group EG2.

Time of Observation	Barbell Bench Press	Lat Pull Down	Dumbbell Squat	Total Load of 3 Exercises
After 6 weeks of intervention	15.32	11.76	16.77	15.50
After 12 weeks of intervention	23.84	23.02	25.41	24.35
After 16 weeks, follow-up period	26.92	25.70	26.30	26.50
*p*-Value	0.00	0.00	0.00	0.00

**Table 2:** Characteristics of the research participants in the aerobic group (EG1), aerobic-resistance group (EG2), and control group (CG).

Index	Group			*p*-Value
	EG1	EG2	CG	
Age [years]	34.21 ± 6.06	37.37 ± 7.08	38.26 ± 7.43	0.20
MetS criterion acc. to IDF	3.07 ± 0.83	3.25 ± 0.86	3.47 ± 0.74	0.30
BMI [kg/m^2^]	34.57 ± 4.58	33.14 ± 4.32	33.20 ± 4.31	0.62
WC [cm]	114.7 ± 10.93	114.8 ± 11.64	115.3 ± 10.54	0.93

MetS—number of metabolic syndrome parameters that meet the criteria of recognition by IDF (International Diabetes Federation), BMI—body mass index, WC—waist circumference, *p*-Value—Kruskal-Wallis test.

**Table 3:** Energy expenditure and energy value of the research participants’ diets in the aerobic group (EG1), aerobic-resistance group (EG2), and control group (CG).

	Gr.	Week 1 Baseline		Week 6 Intervention		Week 12 Intervention		Week 16 Follow-Up		*p*-Value			
		Me	Q1; Q3	Me	Q1; Q3	Me	Q1; Q3	Me	Q1; Q3	F. T. (E.S.)	d 6-1 (E.S.)	d 12-1 (E.S.)	d 16-1 (E.S.)
Energy expenditure [kcal/ week]	EG1	3813.7	3329.3; 4714.2	5697.3	4777.5; 6217.7	5593.5	4495.1; 6249.7	4757.5	4486.6; 6720.4	0.00 (0.70)	0.00 (0.88)	0.00 (0.88)	0.00 (0.83)
	EG2	3746.5	3412.3; 4465.5	5058.9	4419.3; 5903.6	5252.7	4249.9; 5632.8	5520.2	4609.0; 5648.4	0.00 (0.42)	0.00 (0.86)	0.00 (0.86)	0.03 (0.64)
	CG	4465.1	2978.1; 5811.5	3955.1	2760.3; 5497.6	4605.2	3425.4; 5481.4	4682.8	3473.8; 6137.9	0.73 (0.03)	1.00 (0.00)	0.68 (0.13)	0.27 (0.34)
	T.K.	0.90		0.03		0.13		0.60					
Energy value of diet [kcal/day]	EG1	2761.5	2085.8; 3107.0	2445.5	2222.5; 3032.3	2708.5	2327.0; 3336.0	3134.0	2415.5; 3447.5	0.00 (0.32)	0.53 (0.17)	0.22 (0.34)	0.01 (0.66)
	EG2	2669.5	2325.5; 2786.0	2680.5	2531.8; 2996.8	2685.5	2612.3; 3127.0	2900.5	2671.0; 3251.3	0.00 (0.35)	0.03 (0.63)	0.01 (0.83)	0.00 (0.86)
	CG	2689.0	2411.5; 2975.5	2782.0	2653.8; 3055.0	2803.5	2683.8; 3138.3	3112.0	2855.0; 3274.0	0.35 (0.07)	0.27 (0.34)	0.43 (0.24)	0.04 (0.59)
	T.K.	0.81		0.50		0.77		0.95					

EG1—aerobic group; EG2—aerobic-resistance group; CG—control group; d 6-1, d 12-1, d 16-1—differences in results obtained after 6 and 12 weeks of intervention and after 4 weeks of follow-up, respectively, in relation to measurements taken before intervention; Me—median; Q1—lower quartile; Q3—upper quartile; *p* < 0.05—statistically significant difference; *p* ≥ 0.05—statistically insignificant difference; E.S.—effect size; T.K.—Kruskal-Wallis test; F.T.—Friedman test.

**Table 4:** Body composition measured using densitometry in the aerobic group (EG1), aerobic-resistance group (EG2), and control group (CG).

	Gr.	Week 1 Baseline		Week 6 Intervention		Week 12 Intervention		Week 16 Follow-Up		*p*-Value			
		Me	Q1; Q3	Me	Q1; Q3	Me	Q1; Q3	Me	Q1; Q3	T.F. (E.S.)	d 6-1 (E.S.)	d 12-1 (E.S.)	d 16-1 (E.S.)
FFM [kg]	EG1	70.30	67.06; 74.83	70.86	66.40; 73.15	69.80	66.01; 73.33	70.06	66.79; 75.20	0.72 (0.03)	0.24 (0.34)	0.69 (0.13)	0.79 (0.09)
	EG2	67.75	60.39; 73.22	71.82	65.25; 76.00	69.66	63.49; 73.71	67.66	64.61; 74.27	0.00 (0.30)	0.00 (0.89)	0.00 (0.77)	0.00 (0.89)
	CG	67.78	62.69;76.68	68.35	62.63; 77.42	71.25	63.45; 77.44	72.36	65.49; 76.25	0.77 (0.02)	0.74 (0.14)	0.57 (0.22)	0.91 (0.06)
	T.K.	0.46		0.81		0.86		0.79					
BF [%]	EG1	37.40	35.67; 40.25	37.25	35.07; 39.38	38.10	33.80; 39.90	36.95	33.18; 40.25	0.05 (0.18)	0.05 (0.55)	0.03 (0.61)	0.02 (0.60)
	EG2	36.40	34.05; 39.7	35.80	33.80; 38.47	35.00	33.40; 37.80	33.90	33.15; 37.15	0.01 (0.27)	0.01 (0.76)	0.01 (0.79)	0.00 (0.86)
	CG	36.95	34.82; 40.73	37.20	34.53; 42.10	37.55	35.42; 42.12	38.55	36.53; 41.85	0.15 (0.11)	0.16 (0.49)	0.19 (0.46)	0.25 (0.42)
	T.K.	0.87		0.60		0.38		0.26					

FFM [kg]—fat free mass; BF [%]—percentage of body fat; EG1—aerobic group; EG2—aerobic-resistance group; CG—control group; d 6-1, d 12-1, d 16-1—differences in results obtained after 6 and 12 weeks of interventions and after 4 weeks of follow-up, respectively, in relation to measurements taken before intervention; Me—median; Q1—lower quartile; Q3—upper quartile; *p* < 0.05—statistically significant difference; *p* ≥ 0.05—statistically insignificant difference; E.S.—effect size; T.K.—Kruskal-Wallis test; F.T.—Friedman test.

**Table 5:** Concentrations of glucose (GL) and insulin (INS) in participants’ blood in the aerobic group (EG1), aerobic-resistance group (EG2), and control group (CG).

	Gr.	Week 1 Baseline		Week 6 Intervention		Week 12 Intervention		Week 16 Follow-Up		*p*-Value			
		Me	Q1; Q3	Me	Q1; Q3	Me	Q1; Q3	Me	Q1; Q3	T.F. (E.S.)	d 6-1 (E.S.)	d 12-1 (E.S.)	d 16-1 (E.S.)
INS [μIU/mL]	EG1	14.65	10.50; 21.70	10.45	7.60; 16.48	15.05	8.52; 24.3	12.35	8.68; 18.02	0.36 (0.08)	0.23 (0.40)	0.38 (0.31)	0.13 (0.50)
	EG2	16.25	11.20; 22.12	18.75	13.32; 22.18	12.00	8.20; 14.9	10.75	7.66; 12.95	0.03 (0.19)	0.08 (0.56)	0.41 (0.29)	0.03 (0.69)
	CG	20.60	11.15; 22.90	18.70	13.62; 27.25	20.30	13.40; 28.4	16.80	11.80; 28.40	0.12 (0.12)	0.34 (0.34)	0.25 (0.42)	0.91 (0.06)
	T.K.	0.77		0.06		0.06		0.13					
GL [mmol/L]	EG1	5.16	4.75; 5.61	4.96	4.77; 5.16	4.84	4.70; 5.03	4.78	4.64; 5.07	0.12 (0.14)	0.11 (0.39)	0.17 (0.38)	0.02 (0.66)
	EG2	5.13	4.94; 5.30	5.26	5.17; 5.42	5.06	4.86; 5.27	5.00	4.75; 5.20	0.12 (0.12)	0.17 (0.45)	0.73 (0.16)	0.24 (0.42)
	CG	5.31	4.99; 5.48	5.18	5.00; 5.41	4.96	4.84; 5.65	5.14	4.79; 5.38	0.23 (0.09)	0.52 (0.20)	0.20 (0.39)	0.05 (0.57)
	T.K.	0.38		0.08		0.18		0.28					

EG1—aerobic group; EG2—aerobic-resistance group; CG—control group; d 6-1, d 12-1, d 16-1—differences in results obtained after 6 and 12 weeks of interventions and after 4 weeks of follow-up, respectively, in relation to measurements taken before interventions; Me—median; Q1—lower quartile; Q3—upper quartile; *p* < 0.05—statistically significant difference; *p* ≥ 0.05—statistically insignificant difference; E.S.—effect size; T.K.—Kruskal-Wallis test; F.T.—Friedman test.

**Table 6:** Concentrations of irisin (IR) and interleukin-6 (IL-6) in participants’ blood plasma in the aerobic group (EG1), aerobic-resistance group (EG2), and control group (CG).

	Gr.	Week 1 Baseline		Week 6 Intervention		Week 12 Intervention		Week 16 Follow-Up		*p*-Value			
		Me	Q1; Q3	Me	Q1; Q3	Me	Q1; Q3	Me	Q1; Q3	T.F. (E.S.)	d 6-1 (E.S.)	d 12-1 (E.S.)	d 16-1 (E.S.)
IR [ng/mL]	EG1	3.05	2.80; 5.01	2.40	1.34; 11.46	12.56	6.80; 35.31	7.41	4.89; 13.95	0.02 (0.24)	0.06 (0.91)	0.81 (0.18)	0.06 (0.91)
	EG2	10.05	5.50; 28.74	13.14	6.43; 22.90	10.27	5.71; 17.75	10.00	5.66; 19.41	0.69 (0.03)	0.16 (0.58)	0.94 (0.06)	0.58 (0.26)
	CG	8.93	5.07; 12.24	4.53	2.57; 10.50	10.71	6.06; 20.35	12.46	5.23; 19.85	0.05 (0.20)	0.58 (0.26)	0.58 (0.26)	0.30 (0.48)
	T.K.	0.07		0.14		0.99		0.36					
IL-6 [pg/mL]	EG1	17.56	12.59; 22.44	18.98	16.16; 24.95	15.99	14.58; 18.23	14.38	7.94; 15.59	0.01 (0.29)	0.21 (0.40)	0.58 (0.19)	0.10 (0.51)
	EG2	11.79	4.65; 16.40	8.10	2.80; 12.07	5.03	3.10; 9.22	3.10	1.08; 5.17	0.00 (0.32)	0.03 (0.71)	0.01 (0.85)	0.01 (0.85)
	CG	15.19	10.46; 15.99	14.70	12.97; 15.99	15.99	15.39; 17.41	14.38	5.17; 18.43	0.23 (0.09)	0.10 (0.51)	0.07 (0.51)	0.97 (0.02)
	T.K.	0.08		0.00		0.00		0.01					

EG1—aerobic group; EG2—aerobic-resistance group; CG—control group; d 6-1, d 12-1, d 16-1—differences in results obtained after 6 and 12 weeks of interventions and after 4 weeks of follow-up, respectively, in relation to measurements taken before interventions; Me—median; Q1—lower quartile; Q3—upper quartile; *p* < 0.05—statistically significant difference; *p* ≥ 0.05—statistically insignificant difference; E.S.—effect size; T.K.—Kruskal-Wallis test; F.T.—Friedman test.

**Table 7:** Spearman’s rank correlations for participants in the control group (CG), aerobic group (EG1), and aerobic-resistance group (EG2).

	IR EG1 [ng/mL]	IR EG2 [ng/mL]	IR CG [ng/mL]	IL-6 EG1 [pg/mL]	IL-6 EG2 [pg/mL]	IL-6 CG [pg/mL]
Energy value of diet [kcal]	0.23	0.27 *	0.08	−0.05	−0.07	0.06
Energy expenditure [kcal/week]	0.27 *	0.05	0.06	0.35 *	−0.35 *	−0.10
BF [%]	−0.19	−0.11	−0.30 *	0.19	0.38 *	0.05
FFM [kg]	0.35 *	−0.05	−0.06	0.44 *	−0.18	0.22
IR [ng/mL]	1.00	1.00	1.00	0.16	−0.31 *	0.10
IL-6 [pg/mL]	0.16	−0.31 *	0.10	1.00	1.00	1.00
GL [mmol/L]	0.44 *	−0.14	−0.36 *	0.31 *	0.06	−0.12
INS [µIU/mL]	0.37 *	−0.09	−0.42 *	0.36 *	0.22	−0.04

*—statistically significant value p < 0.05; IR EG1—concentrations of irisin in EG1 taken from the four timepoints; IR EG2—concentrations of irisin in EG2 taken from the four timepoints; IR CG—concentrations of irisin in CG taken from the four timepoints; IL-6 EG—concentrations of interleukin-6 in EG1 taken from the four timepoints; IL-6 EG2—concentrations of interleukin-6 in EG2 taken from the four timepoints; IL-6 CG—concentrations of interleukin-6 in CG taken from the four timepoints; FFM—fat free mass; BF—percentage of body fat; GL—glucose level; INS—insulin level.

**Table 8:** Parameters of multiple regression model of the irisin (IR) dependent variable.

Dependent Variable	Parameter Assessment	Standard Error	t Value	*p-*Value
Free parameter	11.83	10.34	1.14	0.25
BF [%]	−0.72	0.21	−3.45	0.00
FFM [kg]	0.43	0.12	3.48	0.00
Dummy: EG2	−6.40	3.08	−2.08	0.04
Dummy: CG	−3.62	2.55	−1.42	0.16

BF—percentage of body fat, FFM—fat free mass, EG2—aerobic-resistance group, CG—control group.

**Table 9:** Parameters of multiple regression model of the IL-6 dependent variable.

Dependent Variable	Parameter Assessment	Standard Error	t Value	*p-*Value
Free parameter	−15.26	7.33	−2.08	0.04
BF [%]	0.25	0.11	2.23	0.03
FFM [kg]	0.19	0.09	2.06	0.04
Dummy: EG2	9.90	1.52	6.51	0.00
Dummy: CG	6.63	1.27	5.21	0.00

BF—percentage of body fat, FFM—fat free mass, EG2—aerobic-resistance group, CG—control group.

## Data Availability

Data are available on request from the corresponding author.

## Supplementary Materials

The following supporting information can be downloaded at: https://www.mdpi.com/article/10.3390/jcm12010369/s1, Table S1: A detailed plan of aerobic-resistance training in aerobic-resistance group (EG2).
