# Supplementary material for: Effect of Exercise Interventions on Irisin and Interleukin-6 Concentrations and Indicators of Carbohydrate Metabolism in Males with Metabolic Syndrome
Source: J Clin Med. 2023 Jan 3;12(1):369. doi: 10.3390/jcm12010369 (PMC9820823; doi:10.3390/jcm12010369)
Supplement: Supplementary file 1 [file jcm-12-00369-s001.zip › jcm-2116045-supplementary.pdf]

Table S1. A detailed plan of aerobic-resistance training in **aerobic-resistance group (EG2).**

	**Trainings 1-3**	**Trainings 4-6**	**Trainings 6<**
Intensity of aerobic training [% HR max]	50	70	70
Duration of aerobic training [min]	20	15	10
Intensity of resistance training [% 1 RM]	50	70	70
Duration of resistance training [min]	30	35	40
Volume of resistance training [exercises x series x repetitions]	3 x 4 x 15	6 x 3 x 12	9 x 3 x 12
Breaks between series [min]	2	1.5	1
Type of training	Whole body training	Training of antagonistic parts	Training of antagonistic parts
Specialised exercises	1. Supported push-ups (Smith machine)<br>2. One arm dumbbell row<br>3. Squads<br>4. Front support	5. Barbell bench press<br>6. Standing dumbbell press<br>7. Bent dumbbell row<br>8. Reverse grip lat pulldown<br>9. Hip thrust lying	10. Cable triceps extension<br>11. Standing dumbbell curl<br>12. Deadlift

1RM – one repetition maximum, HR max – maximal heart rate.
